# A denoising method based on cyclegan with attention mechanisms for improving the hidden distress features of pavement

**DOI:** 10.1038/s41598-023-41212-3

**Published:** 2023-08-25

**Authors:** Lei Liu, Ligang Cao, Congde Lu, Xingtao Yang, Tongbiao Wei, Xiaocui Li, Hengxin Jiang, Lin Yang

**Affiliations:** Key Laboratory of Earth Exploration and Information Techniques of Ministry of Education, Chengdu University of Technology, Chengdu, 610059 Sichuan People’s Republic of China

**Keywords:** Civil engineering, Geophysics

## Abstract

Ground Penetrating Radar (GPR) is one of the most used devices for road structural damages detection. However, due to the different roadbed conditions and various disturbances in the nearby environment during detection, there are great difficulties in interpreting detection images, which also hinders automatic detection based on deep learning. In this work, we design a GPR image denoising method based on Cyclegan. We select the most suitable generator and add different attention mechanisms. After denoising the natural GPR road detection image, using the Yolo (You Only Look Once) to test the accuracy of the original image and the denoised image after adding different attention mechanisms. The detection accuracy is improved by 30%. The results of the detection network and the evaluation of the denoised images by GPR image interpreters indicate that the method has the following advantages: lower requirements for training data sets, a wide range of data sources, low cost, good denoising effect, and automatic detection of GPR images. It is of great help to the automatic detection of GPR images.

## Introduction

The pavement structure is an essential part of the road. Maintaining the stability of the structure helps improve safety and vehicle comfort and reduce fuel consumption. However, pavement structures usually have many defects^1^, including poor interlayers, voids, and looseness. As an important tool for detecting subsurface targets, ground penetrating radar (GPR) has been widely used in engineering due to its convenience, personnel safety, and anti-interference^2,3^. GPR transmits high-frequency electromagnetic waves to the ground and receives reflected waves to bring back objects with different dielectric constants information to achieve the visualization and detection of buried objects. It is a relatively fast technique that provides images of the interior of the overall structure^4,5^. With the rapid development of deep learning, heavy and complex manual detection has been gradually replaced by automatic recognition^6–11^. However, there is a large amount of clutter and noise in the image that is unrelated to the primary signal, affecting the judgment.

Due to the influence of clutter, the effectiveness of various detection networks is limited, and the detection accuracy is usually not high. Currently, most of the main denoising methods are based on ground penetrating radar data: (1) traditional signal transformation methods, such as denoising algorithms based on spatial filtering and transform domain, and (2) statistical feature analysis methods, for example, denoising algorithms based on subspace decomposition. The classic algorithms in spatial filtering algorithms include mean filtering and median filtering^12,13^. After filtering, the image becomes smoother than the original image, which can indeed remove some noise to a certain extent. However, it can also cause signal boundaries to be blurred, resulting in no reduction in detection difficulty; The method of transform domain filtering can more completely separate noise and signals, such as improving continuous wavelet transform and wavelet packet transform from wavelet transform^14^. However, due to the fixed basis of wavelet transform, the denoising results cannot actively adapt to the style of the denoised image, resulting in uncontrollable deviations; The denoising algorithms based on subspace decomposition include Component Analysis (PCA)^15^ and Independent Component Analysis (ICA)^16^, which decompose the data into clutter subspace and target subspace. The division of the two spaces is limited by human experience and there are no fixed standards, so errors are prone to occur.

We use an image-based method, Generative Adversarial Networks (GAN)^17^, to solve problem in denoising. The essence of using Cyclegan to denoise is to transfer the FDTD image style (FDTD image style refer to images without noise) to the measured image to obtain the FDTD style measured image, or the GPR image without noise. Due to the network structure, we do not need paired images and supervised learning. So, much workforce is not required for labeling, and the training process is more straightforward. Cyclegan removes the need for paired training samples by introducing a cycle consistency loss in unsupervised methods^18,19^ that force two mappings to be consistent with each other. Typically, image-to-image translation methods require detecting regions of interest in an input image and learning how to translate the detected regions to the target domain. In unsupervised training, which does not require paired data, it is necessary to learn how to select target regions in an image^20^. In denoising applications, locating the region of interest is more important. Otherwise, the denoising will be incomplete, or the effective signal will be removed as noise. Adding an attention mechanism can solve this problem to a certain extent^21–23^. Attention mechanism can effectively improve the effect of noise reduction. Chen et al.^34^ used extra supervision to train attention networks to improve the quality of overall image conversion. Jie Feng et al.^22^ proposed a symmetric convolutional GAN based on collaborative learning and attention mechanisms to generate high-quality samples for HSI with complex spatial spectral distributions. Xuran Pan et al.^23^ proposed a Generative Adduction network (GAN-SCA) with spatial and channel attention mechanism for robust segmentation of buildings in remote sensing images and the results are superior to several state-of-the-art methods. The above work reflects from different aspects that the effect of deep learning networks is improved after the addition of attention mechanisms.

The detection accuracy of this method is relatively high. Our method has the following characteristics:(1) In the training process of this paper, the measured data sets in production are directly used for training. (2) It is considered that the combination of adding channel attention mechanisms and spatial attention mechanisms at the same time is the most suitable for GPR road structural damages image denoising. (3) We chose the Resnet generator that is more suitable for denoising of road structural damages by GPR.

## Method

The advantage of the proposed network in this paper is that it directly uses the GPR road measured data. It does not need to pre-produce many paired forward simulation data or make a physical model obtain data for training. We add an attention mechanism to the network according to the data features to enhance the denoising effect, improve the imaging quality, and finally improve the detection accuracy for GPR road structural damages. This is a new data processing scheme in GPR road structural damages detection.

### Train generator

**Figure 1:**
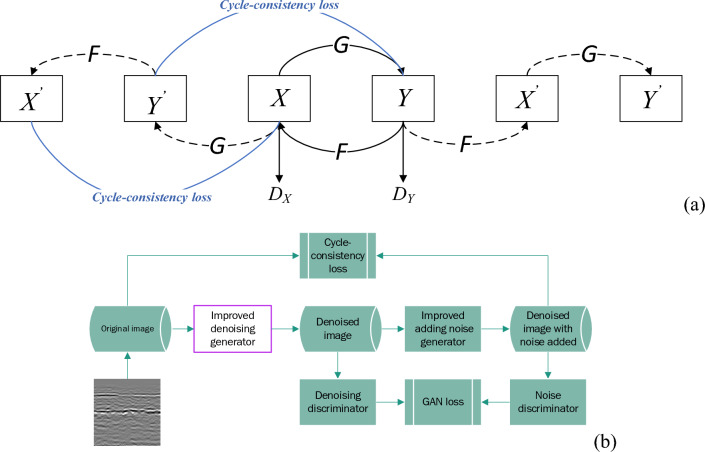
The process of training the denoising generator.

The part we need for training is the denoising generator (purple box in Fig. 1), which is used to convert a noisy road GPR image to a noise-free GPR image. The entire training network includes one denoising generator G_D_, and this generator contains a mapping from a noisy image X to a noise-free (FDTD) image Y; one adding noise generator G_A_, and this generator contains a mapping from a noise-free (FDTD) image Y to a noisy image X; two discriminators D_X_ and D_Y_, used to identify whether the image contains noise, the goals of the two discriminators are different, and the resulting Loss_GAN_ ensures that the generator and the discriminator evolve, thereby ensuring that the generator can produce more realistic pictures. The sum of the loss functions produced by the two discriminators is as follows:

1 $$\begin{aligned} {\text{Loss}}_{{{\text{GAN}}}} = & L_{GAN} \left( {G_{D} ,D_{Y} ,\;X,\;Y} \right) + L_{GAN} \left( {G_{A} ,\;D_{X} ,\;X,\;Y} \right) \\ = & {\mathbb{E}}_{{y\sim p_{data} }} \left( y \right)\left[ {\log D_{Y} \left( y \right)} \right] + {\mathbb{E}}_{{x\sim p_{data} }} \left( x \right)\left[ {\log (1 - D_{Y} \left( {G_{D} \left( x \right)} \right)} \right] \\ & + {\mathbb{E}}_{{x\sim p_{data} }} \left( x \right)\left[ {\log D_{X} \left( x \right)} \right] + {\mathbb{E}}_{{y\sim p_{data} }} \left( y \right)\left[ {\log (1 - D_{X} \left( {G_{A} \left( y \right)} \right)} \right] \\ \end{aligned}$$

$y\sim {p}_{data}$ and $x\sim {p}_{data}$ is data distribution. ${\mathrm{G}}_{\mathrm{D}}$ and ${\mathrm{G}}_{\mathrm{A}}$ are the denoising generator and adding noise generator*.* D_X_ and D_Y_ are two discriminators. X is a noisy image. Y is a noise-free (FDTD) image. ${p}_{data}$ is raw data distribution.

The Fig. 1a shows the network structure, with solid lines representing the path for this training session and dashed lines representing the path for the next training session. Figure 1b shows the overall process. Two generators are trained through the confrontation of two losses, but if we only train with these two losses, the network will not maintain the original image shape and will generate some images similar to the target style; that is, some images will be regenerated Instead of removing noise based on the original image. Therefore, the two different generators should agree but go in precisely opposite directions. A loss needs to be added to ensure that the image before denoising and the image with noise added after denoising is consistent in the same cycle, so a cycle-consistency loss is added between the two generators to ensure that the output image of the generator and the input image styles different, but the same content. The network can use this to obtain the mapping relationship between the two types of images and establish a mapping between the two types of images. The cycle loss function is as follows:

2 $$Loss_{cycle} = {\mathbb{E}}_{{x\sim p_{data} }} \left( x \right)\left[ {\left| {\left| {F\left( {G\left( x \right)} \right) - x} \right||_{L1} \left] { + {\mathbb{E}}_{{y\sim p_{data} }} \left( y \right)} \right[} \right|\left| {G\left( {F\left( y \right)} \right) - y} \right||_{L1} } \right]$$

Mapping function F: Y → X. Mapping function G: X → Y. The L1 distance loss is used to close the reconstructed image to the original input in the L1 sense. The Loss of CycleGAN consists of the above two parts:

3 $$Loss = Loss_{GAN} + Loss_{cycle}$$

### Generator structure

**Figure 2:**
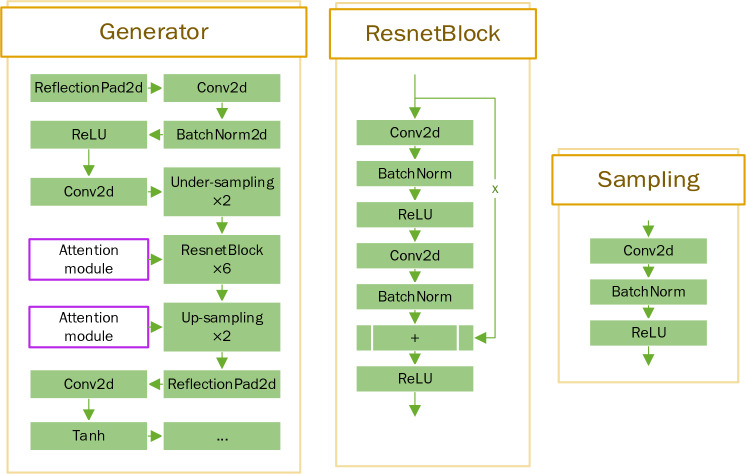
Generator structure (× means there are several identical blocks).

The Fig. 2 shows the structure of upsampling and downsampling. First, the output is mirror-padded, convolved, and normalized. Mirror padding can get better convolution results than padding with a fixed value, and then downsampling, downsampling obtains deeper features by continuously compressing shallow features. After the residual block, the compressed features are upscaled to the size of the original image by upsampling.

### Discriminator structure

**Figure 3:**
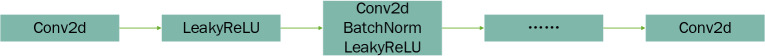
Discriminator structure.

Compared with the discriminator of the original GAN, PatchGAN (The structure is shown in Fig. 3) can take into account the influence of different parts and can better represent the situation of the whole picture. Moreover, because its receptive field corresponds to a small area in the input, this training enables the model to pay more attention to image details, so PatchGAN is often used in scenarios such as high resolution.

### Attention mechanism

**Figure 4:**
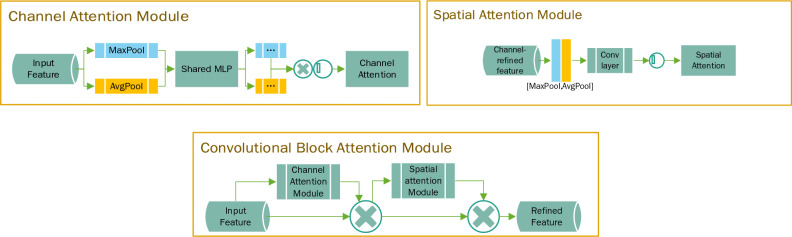
Attention mechanism structure.

Since the GPR image is a single-channel grayscale image, lacking color information compared with the conventional natural image, the information that the image can contain is reduced, and the difference between the target signal and the noise is not apparent. Therefore, the attention module is executed before and after the target signal connection operation, which effectively increases the weight of information learned by the network and improves the denoising effect. This paper uses two attention modules: Channel Attention and Spatial Attention^24–26^. At a high level, the attention module calculates the importance or relevance of each input element by comparing it with other elements in the input sequence. It assigns a weight or attention score to each element, indicating its importance in the context of the current task. These attention scores are then used to compute a weighted sum of the input elements, which serves as the output of the attention module. Channel attention is to weight the channel, and spatial attention is to weight the spatial. Both channel attention and spatial attention can be used independently or in combination. They aim to enhance the understanding and expressive capacity of neural networks, enabling them to adapt better to different tasks and contexts. Four combinations of experiments were conducted in this paper. The attention mechanism is structured as Fig. 4.

## Experiment

**Figure 5:**
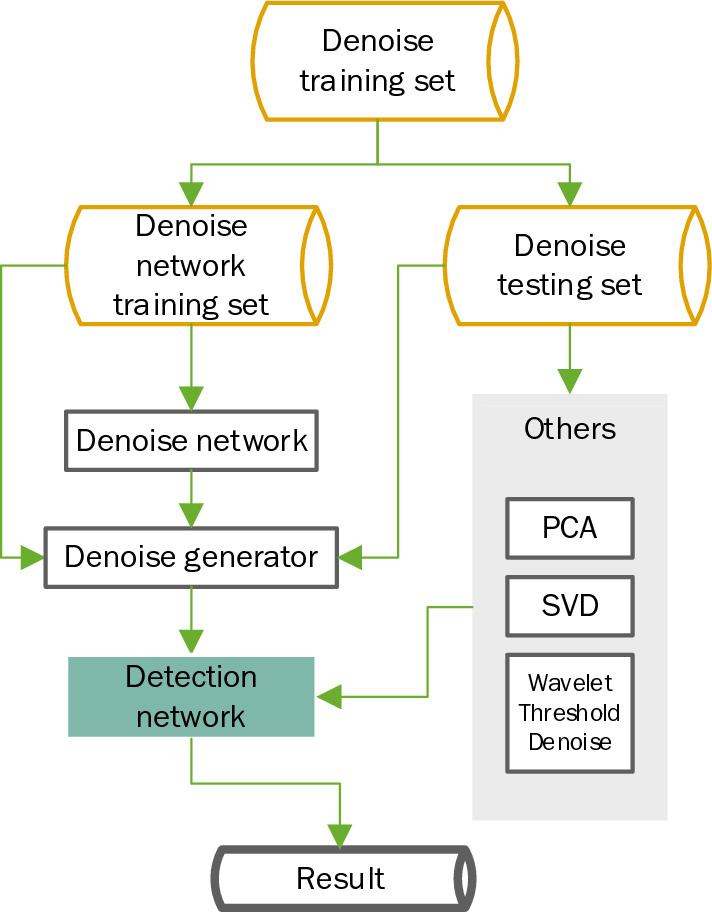
Experimental flow chart.

In the GPR data, the structural damages we need to detect are poor interlayers, voids, looseness, etc., all collectively referred to as structural damages in this paper. We need to remove the noise and clutter that are useless to us except for the structural damages detected in the image. The experimental process is shown in Fig. 5.

The ground-penetrating radar images and FDTD images of the highway are trained by the denoising network proposed in this paper to obtain the generator G, and the data containing the structural damages that needs to be denoised is input into G to obtain the denoised data N, and the denoised data N is obtained. The data N is manually marked with the structural damages and input into the evaluation network test to obtain accuracy and rating radar road structural damages detection.

### Experimental data

**Figure 6:**
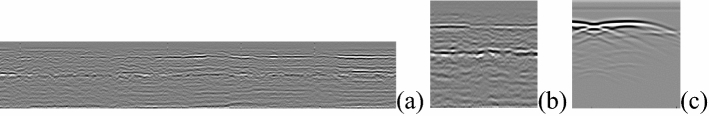
Samples (**a**) Origin data (**b**) Cropped data sample (**c**) No noise (FDTD) sample.

The data used were measured ground penetrating radar images of a certain highway in China and FDTD images produced using GPRMAX^27^ software (arbitrary and reusable). The measured images were collected using a ground penetrating radar instrument of the MALA GX750 model on board, with a speed of 55 km/h and a center frequency of 750 MHz. The coupling method was air coupling. The processed road measured data images and FDTD simulated images are both cropped to the same size and then input. A total of 100 cropped road measured data images form a noise-containing training set, and a total of 100 FDTD simulated images form a noise-free (FDTD) training set. The original data is directly output by the instrument (The resolution is 1110 × 186). The cropping data is square cropped according to the larger value of the disease length and width in the original image. FDTD images are directly output by GPRMAX software (The resolution is 640 × 640). The sample image is shown in Fig. 6.

### Detection network

**Figure 7:**
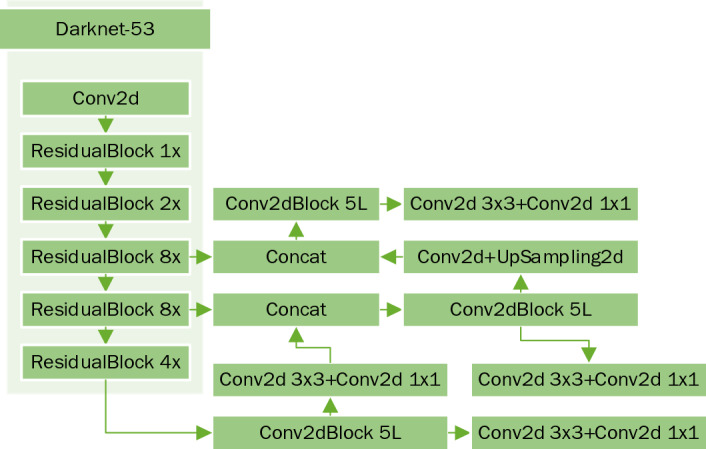
Darknet-53 Structure.

The detection network uses Yolov3, which is a fully convolutional network (FCN). Its authors propose a new feature extraction network, Darknet-53. The Darknet-53 network is shown on the following Fig. 7:

## Results and discussion

### Other denoising methods

**Figure 8:**
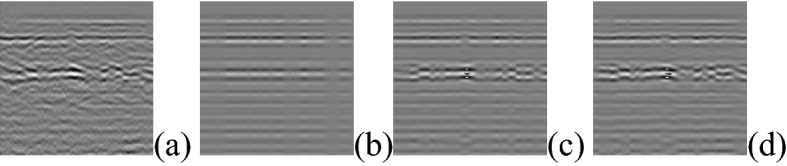
PCA Denoise result (**a**) Original image (**b**) DCI = 30% (**c**) DCI = 40% (**d**) DCI = 50% (DCI = Degree of changing the information).

Principal components analysis (PCA) can handle the sparse noise problem well, but it is an unsupervised method and cannot use label information to increase the recognition rate. It is difficult to determine the number of principal components that need to be maintained in PCA. This becomes difficult when the transformation of the eigenvalues is gentle. This problem exists for the measured GPR data^28^. We indirectly select the retained image's eigenvalues by degree of changing the information. The results are shown in Fig. 8.

**Figure 9:**
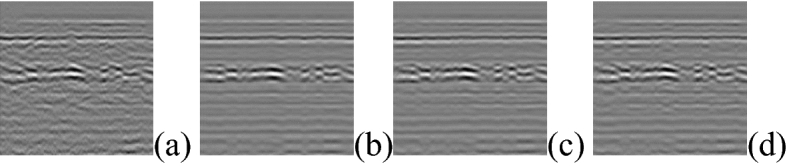
SVD denoise result (**a**) Original image (**b**) k = 3 (**c**) k = 4 (**d**) k = 5 (k is the number of singular values).

Singular value decomposition (SVD) is to decompose the matrix into singular vectors and singular values. Each base contains different discriminative information and reconstruction information, which can be used to extract principal components and remove noise by extracting different components^29,30^. k is the number of singular values. We have experimented with the singular value taken from 1 to 20. The singular value in the front contains more energy, and the latter contains very little energy. The following shows the three values that the denoising effect and the primary structural damages signal remain relatively balanced. The results are shown in Fig. 9.

The basic idea of wavelet threshold denoising proposed by Donoho^31^ is to select an appropriate threshold to remove the noise with lower wavelet coefficients and retain the relatively large wavelet coefficients signal. Wavelet denoising is a synthesis of feature extraction and low pass filtering. A noisy model can be represented as follows:

4 $$S\left( k \right) = f\left( k \right) + \varepsilon \times e\left( k \right),\; k = 0,\;1,\;......,\;n - 1$$

**Figure 10:**
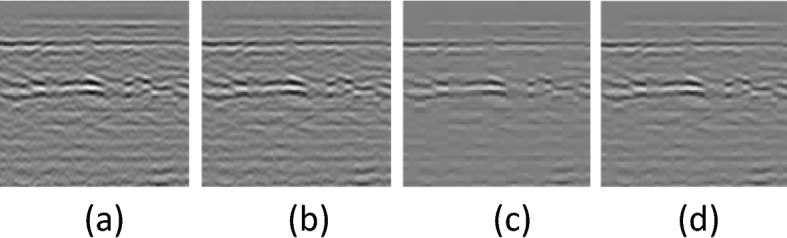
Wavelet threshold denoise result (**a**) Original image (**b**) Threshold is Bayes Shrink (**c**) Threshold is VisuShrink (ε = ε_est_ ) (**d**) Threshold is VisuShrink (ε = ε_est_/2).

In the above formula, $\mathrm{f}\left(\mathrm{k}\right)$ is the useful signal, $\mathrm{S}\left(\mathrm{k}\right)$ is the noise-containing signal, $\mathrm{e}\left(\mathrm{k}\right)$ is the noise, and ε is the standard deviation of the noise figure. We choose the threshold with better effect according to the actual situation^32,33^). The results are shown in Fig. 10.

### Unet generator

**Figure 11:**
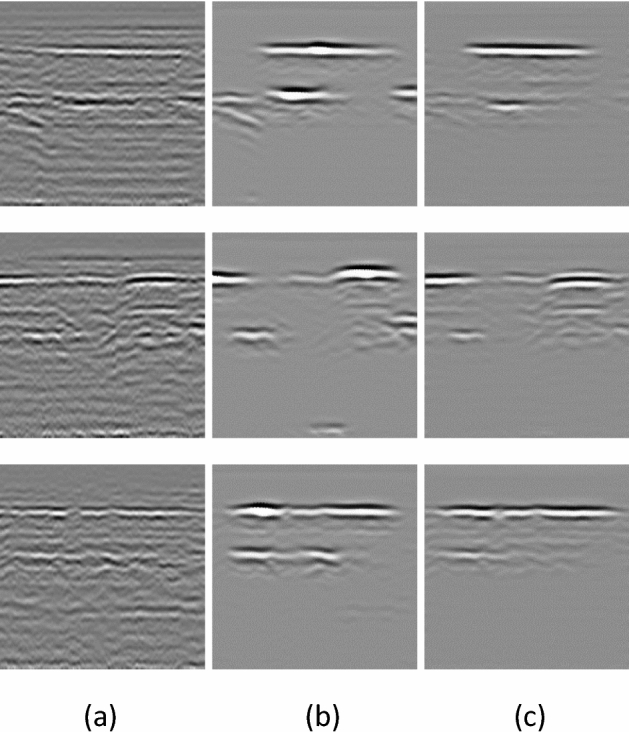
Results of different generators (**a**) Original image (**b**) Results of resnet generator (**c**) Results of unet generator.

From the actual effect in Fig. 11, the Unet generator in the first row of pictures does not completely save the signal in the lower part of the image, and the noise suppression in the middle of the image is not as good as Resnet; The second line signal strength is not as good as the Resnet generator. Based on the above experimental results, the main signals of the Unet generator are not completely preserved, which may easily cause the primary signals to be mistakenly removed when removing noise, which reduces the accuracy of structural damages detection. Therefore, it is recommended to use the Resnet generator for road structural damage denoising.

### PSNR (Peak Signal-to-Noise Ratio)

PSNR is a commonly used indicator for evaluating image quality, which can effectively explain image distortion and is also suitable for evaluating the performance of denoising algorithms. PSNR can evaluate the effectiveness of denoising algorithms by comparing the degree of distortion between the denoised image and the original image. The unit of PSNR is dB. The larger the PSNR value, the less the distortion. MSE has its limitations in describing how similar two images are. Its mathematical formula is as follows:

5 $$MSE = \frac{1}{H \times W}\mathop \sum \limits_{i = 1}^{H} \mathop \sum \limits_{j = 1}^{W} \left( {X\left( {i,\;j} \right) - Y\left( {i,\;j} \right)} \right)^{2}$$

6 $$PSNR = 10 \times \log_{10} \left( {\frac{{\left( {2^{n} - 1} \right)^{2} }}{MSE}} \right)$$

MSE is the mean square error calculated between the original image and the processed image. X and Y are the target images. H and W are the length and width of X and Y. ${2}^{n}-1$ is the maximum numerical value representing the color of image points. If each sampling point is represented by 8 bits, n is 8.

After calculating the PSNR (Table 1), it can be known that adding the attention mechanism can improve the ratio between the maximum signal and the background noise. That is, the background noise can be removed more cleanly, and the signal we need is more prominent. It can be seen from the above table that the PSNR value is the largest when both attention mechanisms are added. This method should be selected if the pursuit of cleaner noise removal.

**Table 1:** PSNR of original images and different processed results.

Method	PSNR/dB
No attention denoise	19.7035
Channel attention denoise	20.6644
Spatial attention denoise	20.0062
Channel attention and spatial attention denoise	21.0392
PCA	21.1426
SVD	21.3158
Wavelet Threshold	20.3212

### SSIM

Structural Similarity (SSIM) is a measure of the similarity between two images. Among the two images used by SSIM, one is the uncompressed undistorted image x, and the other is the distorted image y. SSIM can be obtained as follows:

7 $$SSIM\left( {x,\;y} \right) = \frac{{\left( {2\mu_{x} \mu_{y} + c_{1} } \right)\left( {2\sigma_{xy} + c_{2} } \right)}}{{\left( {\mu_{x}^{2} + \mu_{y}^{2} + c_{1} } \right)\left( {\sigma_{x}^{2} + \sigma_{y}^{2} + c_{2} } \right)}}, \;c_{1} = \left( {k_{1} L} \right)^{2} ,\;c_{2} = \left( {k_{2} L} \right)^{2}$$

${\upmu }_{\mathrm{x}}$ is the mean of x, ${\upmu }_{\mathrm{y}}$ is the mean of y, ${\upsigma }_{\mathrm{x}}^{2}$ is the variance of x, ${\upsigma }_{\mathrm{y}}^{2}$ is the variance of y, and ${\upsigma }_{\mathrm{xy}}$ is the covariance of x and y. ${c}_{1}$ and ${c}_{2}$ are constants used to maintain stability. $L$ is the dynamic range of pixel values. ${k}_{1}=0.01$,${k}_{2}=0.03$. When the two images are identical, the value of SSIM is equal to 1. Humans are not sensitive to the absolute brightness or color of pixels, but very sensitive to the position of edges and textures. SSIM mimics human perception by focusing primarily on edge and texture similarity. The results are shown in Table 2.

**Table 2:** SSIM of original images and different processed results.

Method	SSIM
No attention denoise	0.55927
Channel attention denoise	0.51473
Spatial attention denoise	0.5009
Channel attention and spatial attention denoise	0.50083
PCA	0.6553
SVD	0.69667
Wavelet threshold	0.5997

It can be seen from the table that the SSIM is closest to the original image without the addition of the attention mechanism, and the SSIM is reduced to varying degrees after the attention mechanism is added. Among the several types of added attention mechanisms, the closest to the original image is the one that only added the channel attention mechanism. The reason is that there are many horizontal linear features. Adding a linear attention mechanism is beneficial to maintaining the structural integrity of the graph. The addition of the spatial attention mechanism causes the horizontal linear features to be blurred, resulting in a decrease in the SSIM of the latter two methods. From the denoising point of view, since we remove a lot of structures related to irrelevant signals in the picture, the lower the SSIM, the more signals we remove, and the whole denoising effect is more thorough. We hope that the denoising effect is clean, so choose the method in which both attention mechanisms are added.

### Image analysis

**Figure 12:**
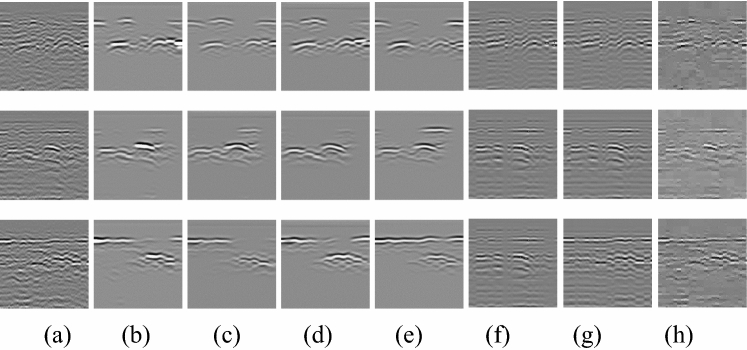
Denoising results of different attention mechanisms (**a**) Original image (**b**) Denoising results without attention mechanism (**c**) Denoising results using channel attention (**d**) Denoising results using spatial attention (**e**) Denoising results using channel attention and spatial attention (**f**) Denoising results of PCA (**g**) Denoising results of SVD (**h**) Denoising results of wavelet threshold.

The Fig. 12a is the original image. As can be seen from Fig. 12b, the denoising effect is already apparent when the attention mechanism is not added, and most of the background noise in the figure is removed, but there is still small clutter outside the target signal. In the second row, because the background clutter is too chaotic, and some noises appear obvious abrupt, it is already somewhat out of tune with the surrounding pictures. It can be seen from Fig. 12c that after adding the Channel Attention mechanism, the stray clutter is significantly reduced, and there is no other stray clutter in the picture except near the main signal. Compared to the denoise without attention mechanisms, it can also be seen that the clutter near the main waveform is also reduced and shrunk to the vicinity of the main waveform. The waveform on the upper right of the second row of graphs is also more apparent, the waveform is not too strong like (b), and the original shape and intensity of the waveform are better maintained. It can be seen from Fig. 12d that the image after adding the Spatial Attention mechanism is relatively compared to Fig. 12b without any attention mechanism added, except that there is basically no other scattered clutter except near the main signal. Compared with adding the Channel Attention mechanism, the waveform is complete and precise, but there are some signals whose strength is not as good as adding the Channel Attention mechanism. The denoising results (Fig. 12e) using Channel Attention and Spatial Attention are cleaner compared to Fig. 12c,d. As can be seen from the first row of images, the signal integrity is better than in Fig. 12c,d. The overall signal strength of the picture is also higher than Fig. 12c, and the surrounding clutter is further shrunk. It can be seen from the second and third row of the picture that the signal in the picture is completely preserved, while the background is cleaner, and the clutter shrinks more thoroughly. The experimental results show that the results of adding Channel Attention and Spatial Attention are the best. Visually speaking, the three columns Fig. 12f,g,h are definitely not as effective as the previous columns.

### Use the annotation of the original image for detection

After manually labeling the structural damages with labeling software, a corresponding JSON file is generated for each image for detection. There are 120 images, including 100 training sets and 20 testing sets, and each image has at least one detection target.

First, we use the annotation of the original image to detect the denoised image. Since the boundary after denoising is more obvious, the indistinguishable boundary information before denoising is highlighted, so the size of the denoised image is different from that of the original image, and the accuracy of using annotation of the original image will be much lower than re-labeling. However, using the original image annotation to detect the accuracy still has a role. It can reflect the structural similarity between the image and the original image to a certain extent. The accuracy results are shown in Table 3.

**Table 3:** The accuracy of using the original image annotation to detect.

Method	AP
Original image	0.579
No attention denoise	0.577
Channel attention denoise	0.642
Spatial attention denoise	0.657
Channel attention and spatial attention denoise	0.617
PCA	0.392
SVD	0.495
Wavelet threshold	0.509

It can be seen from the table that using the annotation of the original image, the accuracy of the image denoised without using the attention mechanism is the highest among all denoising results, indicating that this type of image is the most similar to the original image, followed by the channel attention mechanism. The accuracies are not much different between adding the spatial attention mechanism and adding both. Regarding the accuracy distribution, considering that the image contains a large number of horizontal correlations, the vertical correlation is much less than that of the horizontal, which is probably why the similarity of the spatial attention mechanism is not as high as that of the channel attention mechanism.

But in essence, the target boundary after denoising has changed. For the denoised image, the label of the original image actually has a large error, so AP with proposed method is lower than original image.

### Use the relabeling annotation for detection

**Figure 13:**
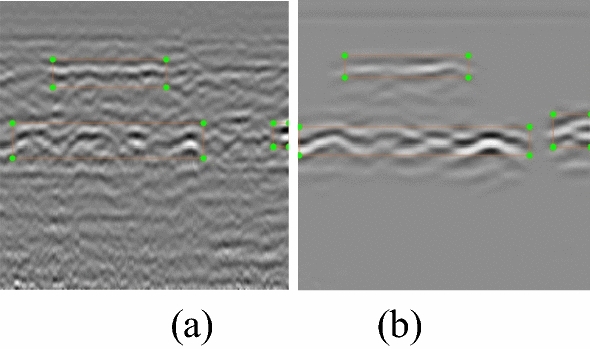
Annotation (**a**) Original image annotation (**b**) Re-labeling annotation.

According to the detection results of the original image label, PSNR, and SSIM, we re-labeled the data in the case of adding two attention mechanisms and re-detected using the detection method described above. The re-labeled labels and detection results are as follows (Annotations are shown in Fig. 13, the accuracy results are shown in Table 4):

**Table 4:** The accuracy of using the re-labeling annotation to detect.

Method	Original image	No attention denoise	Channel attention denoise	Spatial attention denoise	Channel attention and spatial attention denoise
AP	0.617	0.891	0.906	0.758	0.914

**Figure 14:**
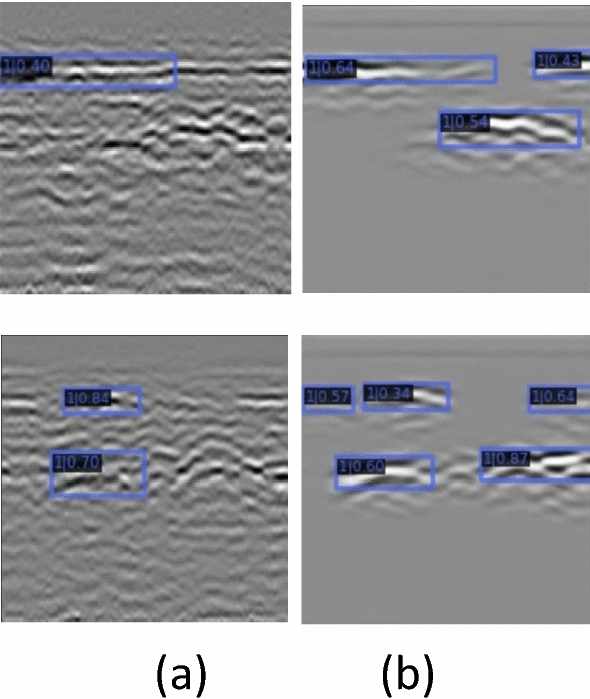
Detection results (**a**) Before denoising (**b**) After denoising.

The actual effect of the detection network before and after denoising is as follows (Fig. 14):

According to the above chart, the image detection accuracy after denoising is significantly improved compared to the original image detection accuracy. The increase is as high as nearly 30% (From 61.7 to 91.4%). From this, it can be seen that the denoising method proposed in this paper has a very obvious effect on improving the accuracy of machine learning detection of structural damages and has considerable practical value.

### The limitation of the work

Network structure can be more streamlined. Because the image itself has only single-channel grayscale data, which is smaller than the amount of natural image data (Colorful images with complex line contours), the detection network can choose a network with a lower number of layers and a simple structure that can speed up the training speed. It will not lead to the lack of deep-level information in training, resulting in a decrease in accuracy.

We can also try whether there are more suitable network modules for ground penetrating radar images, including but not limited to attention mechanisms.

## Conclusion

We have successfully improved the denoising effect of the network used in this article by changing the network structure, adding attention mechanisms, and changing generators. By comparing with traditional methods, the usability of deep learning methods was demonstrated, providing an optional solution for future ground penetrating radar image denoising.

Our method has those advantages: the data sources are more expansive, the acquisition cost is lower, and the processing is closer to reality. The data is conducive to improving the effect, and the experimental results show that selecting the accurate data set in the actual production process is conducive to improving the accuracy, reducing the simulation data production process, and improving work efficiency. Although the related work of other researchers has added attention mechanisms in Cyclegan, they have not discussed the impact of various attention mechanisms. This paper adds channel attention mechanisms and Spatial attention mechanisms in three different combinations, discusses the influence of different combinations on the results. Comparing the results of different generators, according to visual interpretation, the images generated by the Resnet generator are significantly better than those generated by the Unet generator. The detection results confirmed this, and the method improved AP by nearly 30%. At the same time, the trained model runs fast, and in the future, we will apply it to mobile devices to help on-site construction personnel make quick judgments. The following research direction is to adapt this set of methods to other GPR images.

## Data Availability

The datasets generated and/or analysed during the current study are not publicly available due owned by other companies but are available from the corresponding author on reasonable request.
